# Synthesis of Interleukin-10 in Patients with Ulcerative Colitis and *Helicobacter pylori* Infection

**DOI:** 10.1155/2020/4171083

**Published:** 2020-07-06

**Authors:** Jesús K. Yamamoto-Furusho, Gabriela Fonseca-Camarillo, Carlos A. Barrera-Ochoa, Janette Furuzawa-Carballeda

**Affiliations:** ^1^Inflammatory Bowel Disease Clinic, Department of Gastroenterology, Instituto Nacional de Ciencias Médicas y Nutrición Salvador Zubirán, 14080 Mexico City, Mexico; ^2^Department of Immunology and Rheumatology, Instituto Nacional de Ciencias Médicas y Nutrición, Salvador Zubirán, Mexico City, Mexico

## Abstract

**Methods:**

Detection of *H. pylori* infection was performed by a 13C-urea breath test in 31 patients with UC. In each patient, a serum sample was drawn to measure IL-10 by the ELISA technique. Based on the primary breath test result, two groups were formed and serum IL-10 was measured.

**Results:**

Serological IL-10 levels in patients with UC and negative 13C-urea breath test was 10.28 pg/ml whereas in patients with UC and positive 13C-urea breath test was 5.5 pg/ml (*P* = 0.035). IL-10 levels were higher in the inflammatory endoscopic and histological active groups which tested positive in the 13C-urea breath tests for H. pylori (*P* < 0.05).

**Conclusions:**

The role of IL-10 secretion in patients with UC in determining the clinicopathological outcome of infection merits further study. This study suggests an association between serum IL-10 and disease severity in patients with UC and HP infection.

## 1. Introduction

Recent epidemiological evidence suggests an inverse relationship between *Helicobacter pylori* (HP) infection and inflammatory chronic diseases like asthma and eosinophilic esophagitis [1]. HP infection is one of the most widely spread infectious diseases in humans. Also, it has been associated to the presence of the infection with the development of autoimmune diseases such as rheumatoid arthritis, systemic lupus erythematosus, primary biliary cirrhosis, and autoimmune hepatitis [2].

Over the last years, a decrease in the incidence of gastric cancer and peptic ulcer disease and a simultaneous rise in the incidence of inflammatory bowel disease (IBD) have been reported [3–6]. A study carried in Hungary with 133 patients with IBD and a similar control number found a significant inverse relationship with HP infection. Only 13% of the patients with IBD were carriers of the infection whereas in the control group was between 39 and 67% [3]. Another meta-analysis reported that only 27% of patients with IBD had HP against 40% of individuals without the infection suggesting an inverse relationship between both entities [7]. An additional study performed in the US with 1000 IBD patients who had also gastric biopsy also confirmed an inverse relationship between the infection and IBD development [8].

Interleukin 10 (IL-10) is a cytokine implicated in the regulation of human intestinal immune responses. This cytokine acts such as one of most important regulatory interleukins acting in antigen-presenting cells through the inhibition of cytokines and HLA class II [9].

Moreover, IL-10 acts directly in the proliferation and differentiation of T cells [10]. The possible immunological protective effect of HP could be explained because of the virulence factors CagA, VacA, and lipopolysaccharide, which alters Th1 and Th17 responses with the subsequent induction of Treg cells through the expression of Foxp3 in gastric and colonic mucosa [11]. Furthermore, IL-10 was found to be upregulated in colonic mucosa in patients with remission ulcerative colitis (UC) [12].

The aim of this study was to determine the association of HP infection with the synthesis of IL-10 in Mexican patients with UC.

## 2. Patients and Methods

### 2.1. Study Design

This is an exploratory, observational, and cross-sectional study with a total of patients 59 with UC. All UC patients were included during the period from March 2014 to April 2016 belonging to the Inflammatory Bowel Disease Clinic at the Instituto Nacional de Ciencias Medicas y Nutricion.

In order to obtain a significant difference with 80% power, considering a delta of 43% and alpha value of 0.05, a sample size of 50 patients was calculated based upon the results reported from Rudnicka et al. [13]. Details of demographic and clinical characteristics of UC were obtained by a questionnaire, review of records, and personal interview. Disease extension was defined by colonoscopy. The disease activity was determined by the novel integral disease activity index that includes clinical, biochemical, endoscopic, and histological activity [14]. Patients were excluded if they had any concomitant autoimmune disease, obesity, previous HP eradication treatment, current steroid treatment, immunomodulatory o biological agents, or had prior proctocolectomy.

### 2.2. 13-Urea Breath Test

To determine the presence or absence of HP infection, all patients included were asked to undergo 13C-urea breath tests (Infrared Spectrophotometer, BreathTek® UBT Kit, Otsuka America Pharmaceutical). This test was chosen because of its high specificity, sensitivity, and positive and negative predictive values [15]. Patients who had consumed antibiotics or bismuth subsalicylates (at least 4 weeks before the test) or reported prior use of proton pump inhibitor therapy in the past 10 days or use of histamine-blocker 7 days prior to the assessment were excluded from the study. The result of the 13C-urea breath test divided patients into two groups: UC patients with a positive result for HP and UC patients with negative result for HP.

### 2.3. Measurement of IL-10 Serum Levels in Patients with UC

A sample of venous blood (3 ml) was obtained from each subject, collected in an EDTA containing tube, and centrifuged for 10 minutes at 1,000x and immediately stored at -70°C, until used. Serum of each patient (200 *μ*l) was tested in triplicate. The levels of IL-10 were measured in the respective serum samples of the study participants by using a commercial ELISA kit following the manufacturer's instruction (Human IL-10 ELISA MAX™, Pacific Heights Blvd., San Diego, CA, USA). The detection limit for IL-10 was 3.9 pg/ml. The absorbance at 450 nm was recorded using a microplate reader. Our cut-off points for IL-10 serological levels were established based upon the ones reported on the study of Rudnicka et al. [13].

### 2.4. Ethical Considerations

This work was performed according to the principles expressed in the Declaration of Helsinki and approved by the ethical committee from our institution, and a written informed consent was obtained from all subjects.

### 2.5. Statistical Analysis

SPSS Chicago Illinois V. 21 software package was used for statistical analysis. Descriptive data was reported in median and interquartile range. Nonparametric test *U* Mann–Whitney was used to compare IL-10 levels in both groups, and the Fisher exact test in order to evaluate categorical variables. A confidence interval of 95% and a *P* value < 0.05 were considered significant.

## 3. Results

A total of 31 UC patients completed the 13C-urea breath tests and had a sample of venous blood to measure serum IL-10. Eleven patients (34.5%) tested positive in the 13C-urea breath tests for HP, whereas twenty had a negative result (64.5%). Based upon the 13C-urea breath test result, descriptive characteristics are shown in Table 1. No statistical significance was found regarding the result of the breath test and clinical, laboratory, and treatment study variables between both groups, indicating similarity.

### 3.1. IL-10 Serum Levels in Patients with UC

**Figure 1:**
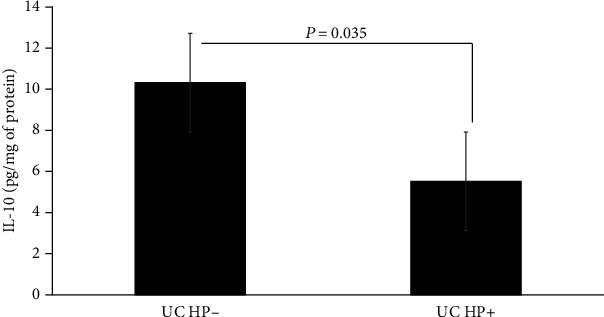
Serological levels of IL-10 in patients with UC in relation to their 13C-urea breath test result.

The median of serological levels of IL-10 in patients with UC who tested negative was 10.28 pg/ml whereas with a positive result was 5.5 pg/ml, this difference was statistically significant (*P* = 0.035), as shown in Figure 1.

In order to evaluate the relation of less studied IL-10 serum levels and colonic inflammation in UC patients, we included histological and endoscopic scores.

**Figure 2:**
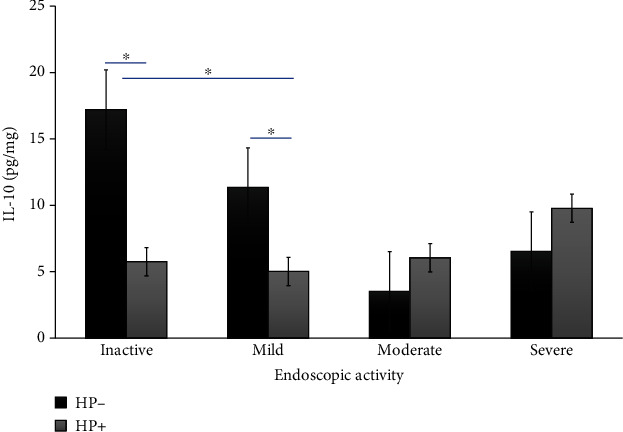
IL-10 levels in ulcerative colitis correlate with endoscopic activity. IL-10 levels in serum from UC patients according to Mayo subscore endoscopic activity; bars show means with standard error of the mean of IL-10, and differences among groups were assessed by the Mann–Whitney *U* test. ^∗^*P* value < 0.05.

**Figure 3:**
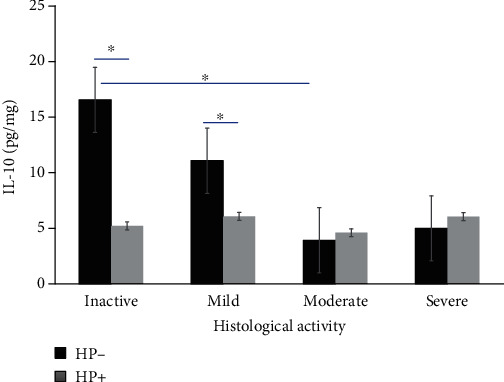
IL-10 levels in ulcerative colitis correlate with histological activity. IL-10 levels in serum from UC patients according to Riley Histological Activity subscore endoscopic activity; bars show means with standard error of the mean of IL-10, and differences among groups were assessed by the Mann–Whitney *U* test. ^∗^*P* value < 0.05.

UC patients with inactive and mild endoscopic and histological activity and HP-negative infection showed some increased levels of IL-10 compared with HP-positive (*P* < 0.05). Conversely, patients with moderate and severe endoscopic and histological activity with UC and HP-positive infection showed some increased levels of IL-10 compared with HP-negative infection (*P* < 0.05). Comparisons among groups are shown in Figures 2 and 3. The IL-10 levels were higher in moderate to severe UC active groups which tested positive in the 13C-urea breath tests for HP.

## 4. Discussion

This study explores the association between HP and IL-10 synthesis in Mexican patients with UC. Epidemiological studies have demonstrated an inverse association between HP infection and the risk of developing IBD. The prevalence of HP infection in Mexico is high and affects around 60-80% of the population [16]. In our study, a larger number of patients tested negative for HP using the urease breath test suggesting a possible protective effect of the HP infection in the development of UC, as other studies have previously reported [17, 18]. The cut-off point for IL-10 serological levels was based from a previous study from Rudnicka et al. [13] who demonstrated that patients with positive urea breath test for HP had significantly higher levels of IL-10 (17.01 ± 9.17 pg/ml) compared to those who had a negative urea breath test (2.75 ± 2.13 pg/ml) (*P* = 0.001).

The IL-10 is an anti-inflammatory cytokine that has been associated with maintaining intestinal homeostasis, though, its targets and complete mechanisms are not completely understood [19]. Previous studies have shown that patients with active IBD had an increased synthesis of IL-10 measured by flow cytometry in CD4+ T cells and B cells. Apparently, elevated IL-10 levels in active disease are triggered by immunological compensatory mechanisms by developing peripheral tolerance through Treg cells suppressing T cell effector responses (Th17, Th1, and Th2) [20, 21].

Zhang et al. discussed the possible mechanism by which HP infection protects against IBD; the authors concluded that HP colonization protects against chronic DSS-induced colitis via balancing Th17/Treg responses and shifting macrophages toward anti-inflammatory M2 phenotype. They found that HP colonization increased Treg cells and IL-10 expression. As to cytokines driving Th17 and Treg differentiation, HP colonization increased TGF*β* and decreased IL-6 and IL-23 [22]. The mechanism of HP involves the role of the dendritic cells and their induction of Treg cells balancing between immunity and infection [23, 24].

An epidemiologic study showed an inverse relationship between seropositive *H. pylori* Cag A+ and the presence of asthma pointing out that this virulence factor may be linked to the immune regulation [25]. The CagA+ virulence factor has been implicated as one of the most important promoters of IL-10 expression in dendritic cells by inhibiting their maturation and producing a tendency for immunological tolerance by suppressing the action of T cells CD4+ and CD8+. The Treg response that occurs in *H. pylori* might be linked to what occurs in IBD as immune responses are exaggerated, and IL-10 is triggered as a compensatory mechanism, to develop peripheral tolerance through Treg and decrease the proinflammatory activity of the disease. Also, the role IL-10 could have is based on genetic regulation because numerous polymorphisms have been associated to the IL-10 synthesis and receptors that may modulate the production of cytokines and may alter the risk of infection of HP [26–29].

Previously, Bodger et al. proposed that IL-10 is implicated in infection and might “damp down” local inflammation in patients with dyspepsia [28]. In patients with UC, the possible mechanism between association of IL-10 and HP infection is to increase levels of IL-10 in patients with moderate and severe activity as a result of immunoregulatory role of IL-10 in intestinal inflammation and triggered by immunological compensatory mechanism in patients with UC.

In our study, we were able to determine that patients with inactive or mild UC and HP infection had lower levels of IL-10 compared to those patients with moderate to severe activity of UC and HP infection who had higher serum levels of IL-10. To our knowledge, this behavior in the expression of IL-10 had not been previously described regarding the clinical activity of the disease. This response is probably based on the fact that HP-stimulated CD19+/IL-10+ B regulator cells and CD11+ dendritic cells (DCs), from the gastric luminal surface and submucosal layer migrates closer to the epithelial surface and retains a semimature phenotype with low MHC II CD80, CD83, CD86, and CD40 expression and low proinflammatory factors secretion (IL-6, IL-12, IL-17, IL-23, etc.). Semimature DCs secrete increased levels of IL-10, TGF-*β*, and IL-18, a process that is required for the differentiation of immunosuppressive Tregs, rather than Th1 or Th17 cells from naive Th0 cells. Through lymphocyte recirculation mechanisms, CD4+/CD25+/FoxP3+ Tregs produced in the gastric mucosa travel to other lymphoid tissues in distant organs to exert a systemic immunoregulatory effect that influences the pathogenesis of various autoimmune and allergic diseases, such as IBD, eczema, and asthma. Moreover, Tregs inhibit the transformation of Th0 cells to Th1/Th17 cells and maintain DCs in a semimature status by direct contact and IL-10 and TGF-*β* secretion. HP-stimulated DCs can subsequently promote Treg differentiation to induce immune tolerance. The weak biological activation of HP by lipopolysaccharides (long 3-hydroxy fatty acids and a deficiency of phosphorylated groups at position 4′ in the D-glucosamine disaccharide backbone of Lipid A) leads to the inefficient activation of NF-*κ*B and production of low levels of proinflammatory molecules. On the other hand, HP can be successfully sensed by TLR2/NOD2 and subsequently activate NLRP3 inflammasome and caspase-1 to promote the maturation of IL-1*β* and IL-18. HP activates NOD2 and ATG16L1 to promote the process of autophagosome formation/autophagy that results in the endocytosis of MHC II and inhibition of NF-*κ*B. Moreover, NOD2 forms trimers with p38 and hnRNP-A1, and the latter subsequently enters the nucleus to stimulate IL-10 transcription. IL-10 and TGF-*β* are required for the activation of the Smad-CDX-MUC2 axis and downstream protective mechanisms, including the inhibition of TLR expression and the NK-*κ*B signaling pathway. Due to the NF-*κ*B independent production mechanism, pro-IL-18 is stably expressed in the cytoplasm and is effectively produced by activated NLRP3 and caspase-1 after HP infection [30, 31]. It is important to mention that this is an interesting clinical finding where HP-positive and moderate to severe activity of UC patients had higher levels of IL-10 compared to HP-negative UC patients with moderate to severe activity suggesting a potential role of HP infection in the production of IL10 as an anti-inflammatory effect especially in patients with moderate to severe UC activity.

Papamichael et al. [32] suggest other possible mechanisms where HP infection could be involved in the pathogenesis of IBD by inducing alterations in intestinal permeability or by causing immunological derangements resulting in absorption of antigenic material and autoimmunity via various immunological pathways.

More studies including a greater number of patients with UC stratified by their disease activity and HP infection are needed to determine the role of IL-10.

Also, the moment of the initial contact with the infection was not considered and cytokine and immune responses may vary at different times on the development of IBD and therefore, the length of the infection might impact IL-10 levels.

The presence of inflammatory activity in UC may have important consequences in the synthesis of IL-10 and, therefore, may impact the development of chronic colonization of HP.

This study has some limitations. First, we included a small number of patients based on the rigorous inclusion criteria to select patients. Second, all the patients were under treatment with 5-aminosalicylate which could alter IL-10 production. Third, a control group of healthy individuals without and with HP infection would have been desirable. Moreover, in this study, it is not possible to establish the mechanism how HP infection induces IL-10 production.

In conclusion, this is the first study to explore HP infection and serum IL-10 synthesis in patients with active and remission UC. The IL-10 showed an association with disease severity in patients with UC and HP infection.

## Figures and Tables

**Table 1:** Clinical and demographic characteristics of ulcerative colitis patients divided based upon urea breath test result.

Characteristics	13C-urea breath test Positive (*H. pylori*+)		13C-urea breath test Negative (*H. pylori*-)		*P* value ≤ 0.05
	No. of patients *n* = 11	Frequency (%)	No. of patients n =20	Frequency (%)	
Onset of disease (years-range)	34 (24-52)	—	28 (17-62)	—	0.14
Mean age (years-range)	42 (34-68)	—	39 (23-69)	—	0.33
Disease duration (years-range)	10 (4-18)	—	10 (3-35)	—	0.64
Body mass index (kg/m^2^-range)	24.4 (20.8-29.9)	—	23.8 (18.8-29.9)	—	0.50
Gender					0.61
Male	4	34	7	35	—
Female	7	64	13	65	—
Relapse pattern					0.37
Rare (≤1/year)	10	91	14	70	
Common (≥2 relapses/year)	1	9	4	20	
Continuous activity (persistent symptoms of active UC without remission period)	0	0	2	10	
Disease extension					0.19
Extended colitis	8	73	12	50	
Left colitis	0	27	1	5	
Proctosigmoiditis	3	0	7	35	
Endoscopic activity					0.60
Inactive	2	18	5	20	
Mild	4	36	9	50	
Moderate	5	46	3	15	
Severe	—	—	3	15	
Histological activity					0.28
Inactive	4	33	4	20	
Mild	4	36	10	50	
Moderate	2	18	5	25	
Severe	1	9	1	5	
Therapy with 5-aminosalicylate					0.27
≤2 g	7	63	7	35	
>2 g	4	36	13	65	
Laboratories					
Hemoglobin (g/dL-range)	14.6 (13.5-17.1)	—	13.9 (11.4-17.5)	—	0.10
Hematocrit (%-range)	44.4 (38.4-50.5)	—	42 (34.7-53.4)	—	0.37
Leukocytes (×10^3^-range)	6.1 (3.9-9.6)	—	6.7 (4.8-10.6)	—	0.72
Platelets (K/ul-range)	270 (136-369)	—	280 (201-372)	—	0.53
Albumin (g/dl-range)	4.6 (4.20-5.0)	—	4.3 (3.6-4.8)	—	0.15
ESR (range)	2 (0-11)	—	6.5 (2-59)	—	0.39
High sensitivity CPR (range)	0.12 (0.04-0.26)	—	0.23 (0.02-2.13)	—	0.17

## Data Availability

All data and figures used to support the findings of this study are included within the article.
